# Ice swimming – ‘Ice Mile’ and ‘1 km Ice event’

**DOI:** 10.1186/s13102-015-0014-9

**Published:** 2015-09-03

**Authors:** Beat Knechtle, Thomas Rosemann, Christoph A. Rüst

**Affiliations:** 1 Gesundheitszentrum St. Gallen, Vadianstrasse 26 9001, St. Gallen, Switzerland; 2 Institute of Primary Care, University of Zurich, Zurich, Switzerland

## Abstract

**Background:**

Ice swimming for 1 mile and 1 km is a new discipline in open-water swimming since 2009. This study examined female and male performances in swimming 1 mile (‘Ice Mile’) and 1 km (‘1 km Ice event’) in water of 5 °C or colder between 2009 and 2015 with the hypothesis that women would be faster than men.

**Methods:**

Between 2009 and 2015, 113 men and 38 women completed one ‘Ice Mile’ and 26 men and 13 completed one ‘1 km Ice event’ in water colder than +5 °C following the rules of International Ice Swimming Association (IISA). Differences in performance between women and men were determined. Sex difference (%) was calculated using the equation ([time for women] – [time for men]/[time for men] × 100). For ‘Ice Mile’, a mixed-effects regression model with interaction analyses was used to investigate the influence of sex and environmental conditions on swimming speed. The association between water temperature and swimming speed was assessed using Pearson correlation analyses.

**Results:**

For ‘Ice Mile’ and ‘1 km Ice event’, the best men were faster than the best women. In ‘Ice Mile’, calendar year, number of attempts, water temperature and wind chill showed no association with swimming speed for both women and men. For both women and men, water temperature was not correlated to swimming speed in both ‘Ice Mile’ and ‘1 km Ice event’.

**Conclusions:**

In water colder than 5 °C, men were faster than women in ‘Ice Mile’ and ‘1 km Ice event’. Water temperature showed no correlation to swimming speed.

## Background

Official open-water ultra-distance swimming races at the world class level have been held since 2000 [1–3] apart from the ‘English Channel Swim’ which has been held as a solo swim since 1875 [4].

Sex differences are lower in swimming compared to running [5]. In elite pool swimmers competing in breaststroke, backstroke, medley and freestyle up to 1500 m, women reduced the gap to men with increasing race distance [6–10]. In long-distance open-water swimming from 5 to 25 km, women reduced the gap to men in 10 km, but not in 25 km [3]. In longer distances, however, women were able to reduce the sex difference in races such as ‘La Traversée Internationale du Lac St-Jean’ held in Canada between 1955 and 2012 [11] and ‘Maratona del Golfo Capri-Napoli’ held in Italy from 1954 to 2013 [12].

In open-water swimming, women are also able to beat men [13, 14]. Two recent studies investigating female and male open-water swimmers described for the first time that women were able to swim faster than men [13, 14]. In the 46-km ‘Manhattan Island Marathon Swim’ held in water temperatures below +20 °C, the best women were ~13 % faster than the best men [13]. It was argued that the low water temperature and the higher body fat in women were the most likely reasons that women were able to beat men [13]. Similarly to the ‘Manhattan Island Marathon Swim’, the fastest women crossed the ‘Catalina Channel’ faster than the fastest men [14].

Women might outperform men in open-water swimming due to greater proportions of body fat – *e.g.,* better buoyancy or insulation. Female swimmers have more adipose tissue than male swimmers [15] and the distance between the centre of buoyancy and the centre of mass is larger for men compared to women [15]. Both the centre of buoyancy and mass are more caudal in women compared to men [15]. Higher body fat (*i.e.,* thicker skinfold thicknesses and more subcutaneous fat) helps to endurance longer in cold water [16–18]. It has been shown in several instances that subjects with higher body fat were able to stay longer in cold water [16–18]. The longer swimming times of subjects with higher body fat were attributed largely to their greater buoyancy enabling them to keep their heads above water during the early hyperventilation [19].

Ice swimming is a new discipline within open-water swimming. In ice swimming, Lynne Cox [20] and Lewis Pugh [21] are the two pioneers with the first and most outstanding achievements. Lynne Cox swam in 1975 the 16-km Cook Strait in New Zealand at 10 °C, in 1987 the Bering Strait from the island of Little Diomede (Alaska) to Big Diomede (former Soviet Union) in 2:05 h:min at 6–7 °C and in 2002 in Antarctica from the ship ‘Orlova’ to Neko Harbor 1.96 km in 25:00 min:sec [20]. Lewis Pugh swam in 2007 across the Geographic North Pole for 1 km at −1.7 °C in 18:50 min:sec, and in 2010 1 km in 2 °C for 22:51 h:min across Lake Pumori, a glacial lake on Mount Everest at 5’300 m above sea level [21].

Based upon these achievements of Lynne Cox [20] and Lewis Pugh [21], Ram Barkai from South Africa founded in 2009 the ‘International Ice Swimming Association’ (IISA) [22]. The IISA introduced the ‘Ice Mile’ as its ultimate achievement of swimming in ice cold water. An ‘Ice Mile’ is defined as swimming 1 mile (1.609 km) in water of +5 °C or colder [22]. The swimmer must complete the mile unassisted using only a pair of swimming goggles, a swimming cap and a standard swimming suit [22]. The IISA has defined how an ice swimmer can complete an official ‘Ice Mile’ [23]. Following the IISA, the swim distance must be at least 1 one mile or 1609.3 m [23]. The swim must be in water of a temperature of 5 °C or lower and measured as follows [23]: The water temperature must be measured for at least 10 min between 127 and 508 mm below the water surface, the water temperature must be established by using the average reading obtained from three digital thermometer readings with a temperature accuracy of ±1 °C. The thermometers must be water submerged thermometers and no laser or infra-red thermometers are allowed. The official water temperature must be measured no more than 30 min before the start of the swim. The swim must be followed by independent observers [23]. In 2014, IISA introduced the ‘1 km Ice event’ covering 1 km in addition to the ‘Ice Mile’. The event allows for swimmers to compete in icy waters of +5 °C or less under IISA rules for 1000 m. Swimming times in ‘Ice Mile’ and ‘1 km Ice event’ are recorded since 2009 and 2014, respectively [22].

Little is known about swimming performance in ice cold water; one study regarding performance of two men [16] and no published data regarding performance of women exists. The present study investigated the performances for female and male swimmers in ‘Ice Mile’ and ‘1 km Ice event’ since 2009. The first man completing an official ‘Ice Mile’ was Ram Barkai on January 31, 2009, in Lake Zurich, Switzerland, in 43:00 h:min. The first woman achieved an ‘Ice Mile’ on July 23, 2011, in Fraserburg, South Africa in 26:45 h:min. Although the swim time for the women was faster than the time for the man, Ram Barkai was swimming 2.2 km, longer than the official distance of an ‘Ice Mile’. Based upon the fact that Lynne Cox was the first person to swim in icy water [20] before the first man [21] and the recent findings that women were able to beat men in open-water swimming in low water temperatures (<20 °C) [13, 14], we hypothesized that women would be faster than men in both ‘Ice Mile’ and ‘1 km Ice event’. We also considered repeated finishes in our analysis since it was hypothesized that extensive previous experience was important for successful ice swimming [16, 17].

## Methods

### Ethics

All procedures used in the study were approved by the Institutional Review Board of Kanton St. Gallen, Switzerland with a waiver of the requirement for informed consent of the participants given the fact that the study involved the analysis of publicly available data.

### Data sampling and data analysis

The data set for this study was obtained from the website of the ‘International Ice Swimming Association’ (IISA) [22]. The last access to the database was March 29, 2015. On the IISA website, the results of successful swims are presented with date, name of the athletes, sex, water temperature, and wind chill. Performance time was extracted from the database. Since some ‘Ice Miles’ were longer than one mile, we converted all performance times in ‘Ice Mile’ and ‘1 km Ice event’ to swimming speed in km/h.

### Statistical analysis

Each set of data was tested for normal distribution (D’Agostino and Pearson omnibus normality test) and for homogeneity of variances (Levene’s test) before statistical analyses. Sex difference in percent (%) was calculated using the equation ([swim time for women] – [swim time for men]/[swim time for men] × 100). To find differences between two groups, a Student’s *t*-test was used in case of normal distributed data (with Welch’s correction in case of unequal variances) and a Mann–Whitney test was used in case of not normal distributed data. A mixed-effects regression model with swimmer as random variable to consider swimmers who completed several events was used to analyse changes in swimming speed across years. We included sex, experience (*i.e.,* number of previous successful swims), water temperature, wind chill and calendar year as fixed variables. We also considered interaction effects between sex, experience, water temperature and wind chill. The final model was selected by means of Akaike information Criterion (AIC). A potential correlation between water temperature and swimming speed was tested using Pearson correlation coefficients. Statistical analyses were performed using IBM SPSS Statistics (Version 22, IBM SPSS, Chicago, IL, USA) and GraphPad Prism (Version 6.01, GraphPad Software, La Jolla, CA, USA). Significance was accepted at *p* < 0.05 (two-sided for *t*-tests). Data are given as mean ± standard deviation (SD).

## Results

### ‘Ice Mile’

**Fig. 1:**
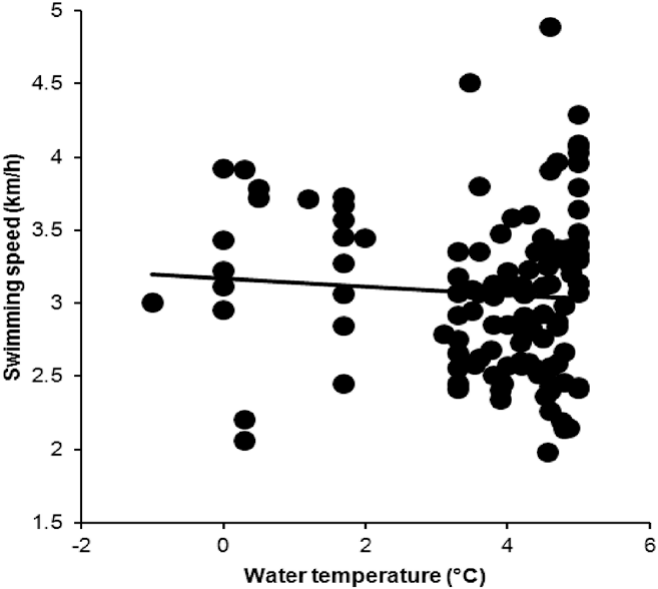
Correlation between water temperature and swimming speed for men in ‘Ice Mile’

**Fig. 2:**
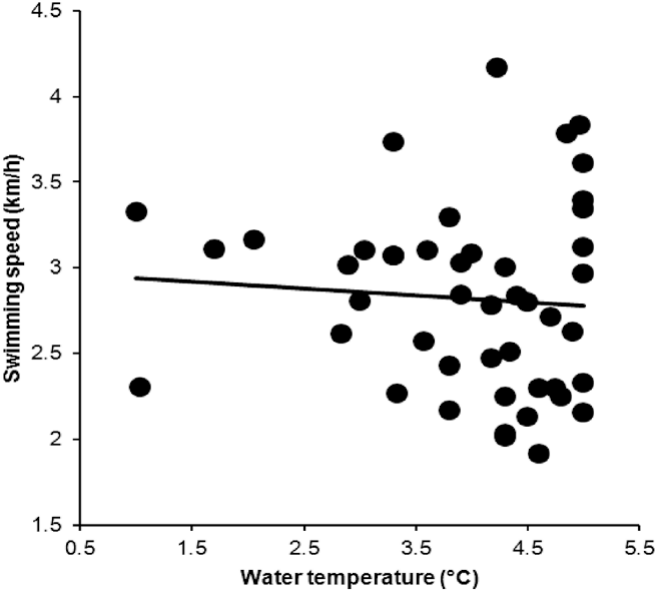
Correlation between water temperature and swimming speed for women in ‘Ice Mile’

**Fig. 3:**
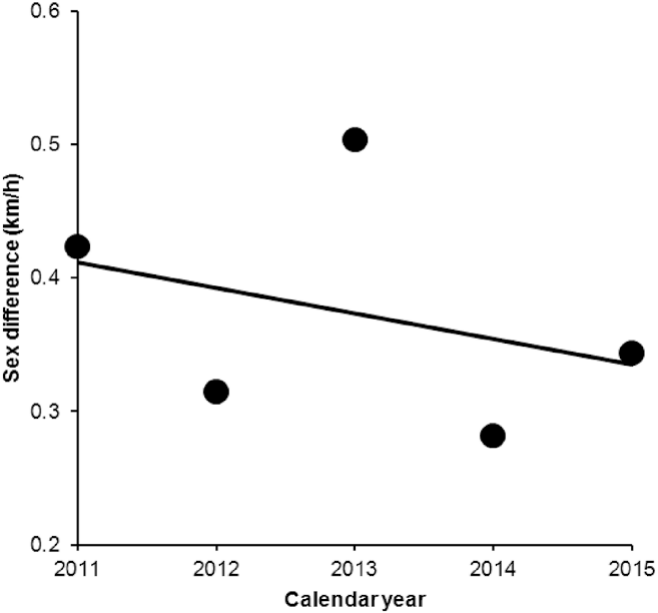
Sex difference between the annual fastest women and men in ‘Ice Mile’ between 2011 and 2015

From 2009 to 2015, data from 169 swimmers (123 men and 46 women) were available. Men were faster than women for the three fastest, the ten fastest and when all women and men were compared (Table 1). Water temperature in ‘Ice Mile’ was +3.76 ± 1.39 °C, varying from −1.0 to +5.0 °C. Water temperature and swimming speed showed no correlation for men (*r*^*2*^ = 0.0037, *p* = 0.520, Fig. 1) and women (*r*^*2*^ = 0.0288, *p* = 0.307, Fig. 2). Calendar year, number of attempts, water temperature and wind chill were not associated with swimming speed for women and men (Table 2). Swimming speed showed no changes across calendar years for men (*r*^*2*^ = 0.19, *p* = 0.329) and women (*r*^*2*^ = 0.70, *p* = 0.077). The sex difference in swimming speed between the annual fastest women and men remained unchanged (*r*^*2*^ = 0.12, *p* = 0.576) between 2011 and 2015 where men were 0.37 ± 0.09 km/h (17.9 ± 5.3 %) faster than women (Fig. 3).

**Table 1:** The fastest women and men with sex difference for ‘Ice Mile’ and ‘1 km Ice event’

‘Ice Mile’					
Women (km/h)		Men (km/h)	Difference (km/h)	Difference (%)	Significance
Three fastest	3.92 ± 0.21	4.56 ± .030	0.63 ± 0.11	16.1 ± 2.3	*p* = 0.010
Ten fastest	3.56 ± 0.31	4.16 ± 0.31	0.59 ± 0.12	16.9 ± 4.0	*p* < 0.0001
All	2.82 ± 0.53	3.06 ± 0.54	0.24 ± 0.01	8.5 ± 1.8	*p* < 0.0001
‘1 km Ice event’					
Three fastest	4.02 ± 0.13	4.44 ± 0.15	0.41 ± 0.50	10.3 ± 0.8	*p* = 0.0024
Ten fastest	3.53 ± 0.36	4.25 ± 1.55	0.71 ± 0.22	21.1 ± 8.3	*p* < 0.0001
All	2.92 ± 0.58	3.39 ± 0.57	0.47 ± 0.01	13.8 ± 1.7	*p* < 0.0001

**Table 2:** Coefficients and standard errors (SE) from a multivariable regression model. Interactions between variables are also presented

Parameter	Estimate	SE	df	*t*	*p*-value
Constant term	30.61	22.39	141.26	1.36	0.17
Female	0.23	0.17	129.30	1.29	0.19
Calendar year	−0.01	0.01	141.25	−1.32	0.18
Number of attempts	0.007	0.01	82.60	0.62	0.53
Water temperature	−0.005	0.01	59.42	−0.48	0.63
Wind chill	0.002	0.002	82.47	0.86	0.38
Female × Number of attempts	−0.12	0.09	61.70	−1.31	0.19
Female × Water temperature	−0.04	0.03	148.47	−1.40	0.16
Female × Wind chill	−0.03	0.03	143.75	−1.07	0.28
Water temperature × × Wind chill	0.0003	0.001	75.83	0.31	0.75
Female × Water temperature × Wind chill	0.006	0.008	149.11	0.80	0.42
Female × Number of attempts × Water temperature × Wind chill	0.002	0.002	55.64	0.95	0.34
Female × Number of attempts × Water temperature × Wind chill	−8.5 × E^−5^	0.0005	37.5	−0.16	0.87

### ‘1 km Ice event’

**Fig. 4:**
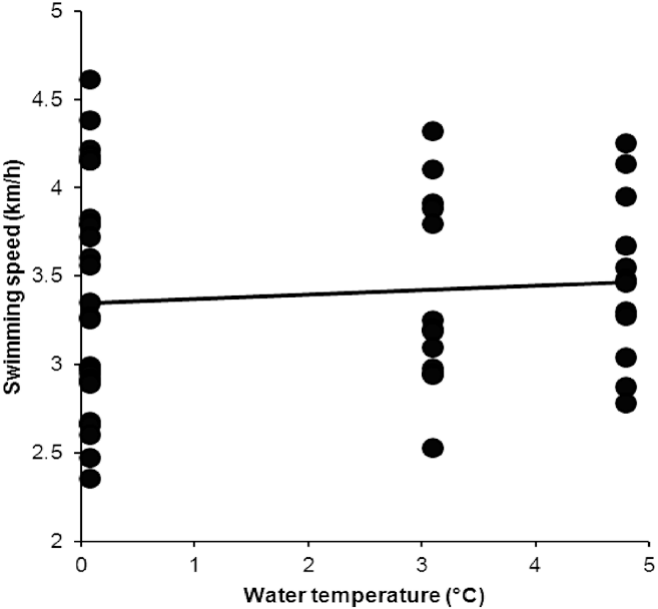
Correlation between water temperature and swimming speed for men in ‘1 km Ice event’

**Fig. 5:**
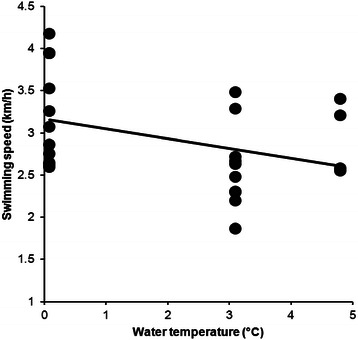
Correlation between water temperature and swimming speed for women in ‘1 km Ice event’

Data from 78 swimmers (53 men and 25 women) were available. All data were from winter 2015; therefore, no analyses for trends across calendar years were possible. Men were faster than women for the three and the ten fastest (Table 1). Water temperature in ‘1 km Ice event’ was +3.79 ± 0.84 °C, varying from +3.1 °C to +4.8 °C. Water temperature and swimming speed were not associated in men (*r*^*2*^ = 0.094, *p* = 0.306, Fig. 4) and women (*r*^*2*^ = 0.023, *p* = 0.452, Fig. 5).

### Sex difference

We compared the sex difference between ‘Ice Mile’ and ‘1 km Ice event’ for the fastest swimmers. For the three fastest in ‘Ice Mile’ and ‘1 km Ice event’, the sex differences were 16.1 ± 2.3 % and 10.3 ± 0.8 %, respectively. The difference of 5.8 ± 1.6 % between the two events was statistically significant (*p* = 0.024). For the ten fastest in ‘Ice Mile’ and ‘1 km Ice event’, the sex differences were 16.9 ± 4.0 % and 21.1 ± 8.3 %, respectively. The sex difference of 4.2 ± 7.1 % for the ten fastest in ‘1 km Ice event’ and ‘Ice Mile’ was not statistically significantly different (*p* > 0.05).

## Discussion

This study investigated the performance and the sex difference in performance for women and men completing an official ‘Ice Mile’ and ‘1 km Ice event’ with the hypothesis that women would be faster than men in ‘Ice Mile’ and ‘1 km Ice event’. The main findings were, (*i*), the fastest men were faster than the fastest women in ‘Ice Mile’ and ‘1 km Ice event’, (*ii*), the sex difference remained unchanged across calendar years in ‘Ice Mile’ and, (*iii*), no correlation existed between water temperature and swimming speed for women and men in ‘Ice Mile’ and ‘1 km Ice event’.

### The fastest men were faster than the fastest women

A first important finding was that women were not faster than men as hypothesized. Based upon previous findings where women were faster than men in the ‘Manhattan Island Marathon Swim’ [13] and the ‘Catalina Channel Swim’ [14] where athletes competed at water temperatures < 20 °C we hypothesized that women would be faster than men in swimming in ice cold water. However, the fastest men were faster than the fastest women in both ‘Ice Mile’ and ‘1 km Ice event’. We based our assumption that women would be faster than men in ice swimming on previous reports that female open-water ultra-distance swimmers have more body fat than male swimmers [24]. A study investigating body composition in open-water swimmers in the 26.4-km ‘Marathon Swim’ in Lake Zurich, Switzerland, showed that men (18.8 ± 4.5 % body fat) had ~12 % less percent body fat than women (30.7 ± 3.7 % body fat) [24].

Most probably, percent body fat is not the most important predictor for a fast swim time in open-water swimming. In female and male open-water ultra-endurance swimmers in the 26.4-km ‘Marathon Swim’ in Lake Zurich, Switzerland, a potential association between anthropometric characteristics and race time was investigated [25]. For men, body height, Body Mass Index (BMI), length of arm, and swimming speed during training were related to race time after bivariate analysis. For women, swimming speed during training was associated with race time. In the multivariate analysis, BMI and swimming speed during training were related to race time for men [25]. Body fat percentage was not predictive for women and men in open-water distance swimming at moderate water temperatures (>20 °C) [25] and also in ice swimming.

Another potential explanation could be differences in body core temperature between women and men during open-water distance swimming [26]. In a 10-km open-water swimming competition held at a water temperature of ~21 °C, the drop in body core temperature was more pronounced in women (4.2 ± 0.7 °C) than in men (2.7 ± 0.8 °C) [26]. In men, race time was inversely related to pre-competition body temperature [26]. Differences in body composition between women and men might be of importance [18, 27]. In a 19.2-km open water swimming race, longer race duration was associated with an increased risk of hypothermia, and higher BMI was associated with a decreased risk of hypothermia [27]. In swimmers competing in water temperature below 11 °C, athletes with lower body fat finished their swims after less time in the water than those with thicker skinfold thickness. The rectal temperatures were, however, not significantly lower [18]. In the present study investigating women and men swimming 1 mile and 1 km in water below 5 °C, the duration of the swims was rather short and body core temperature might not have dropped seriously.

### Sex difference in performance

The first woman completed in 2011 an official ‘Ice Mile’. Between 2011 and 2015, the sex difference in the annual fastest women and men in ‘Ice Mile’ remained unchanged at ~18 %. For the three and the ten fastest in ‘Ice Mile’, the sex difference was ~16–17 %. For ‘1 km Ice event’, however, the sex differences for the three and ten fastest were ~10 % and ~21 %, respectively.

The sex difference in swimming speed has been investigated in several studies for pool [5, 28–31] and open-water [1, 11–13, 32–34] swimmers. In master pool swimmers aged from 35 to 70 years, the percent sex difference in freestyle swimming from 50 m to 1500 m became progressively smaller with increasing distance from 50 m (19 ± 1 %) to 1500 m (11 ± 1 %) [5]. When swimming performances in different disciplines and strokes were investigated, men were generally ~8.9 % faster than women [31]. Although two very recent studies showed that women were able to beat men in open-water swimming [13, 14], men were generally faster than women in pool [6, 29] and open-water [1, 11, 12, 33, 34] swimming. However, in some studies, women were able to achieve similar performances to men in open-water swimming [28, 32].

The sex differences in these ice swimmers were higher compared to sex differences reported for pool [6, 29] and open-water [1, 3] swimmers competing at moderate water temperatures (~20 °C). For pool swimmers competing at world-class level (*i.e.,* finalists in Olympic Games and World Championships) in different strokes and distances up to 1500 m, the sex difference was at ~9–10 % [6]. For freestyle swimming from 50 m to 1500 m, the sex difference was unchanged at ~9 % [29]. In elite 10-km open-water swimmers competing at world-class level, the sex difference was at ~7 % [1]. In the annual fastest swimmers competing in FINA World Cup races from 2000 to 2012 in 5 km, 10 km and 25 km, the sex differences in swimming speed remained at ~7 %, ~6 % and ~9 %, respectively [3]. The sex difference for the three fastest ice swimmers (~10 %) was comparable to the findings for freestyle pool swimmers up to 1,500 m [29], but increased to ~21 % for the ten fastest ever. Differences in body composition [24] and body core temperature [26] between women and men might explain these differences.

Between 2011 and 2015, the annual fastest men were ~18 % faster than the annual fastest women. Women reduced the gap to men in open-water ultra-distance swimming in the last years [11, 12]. The short period of five calendar years was the most likely reason for the unchanged sex difference. Other studies investigated time periods of decades where women considerably reduced the gap to men [11, 12, 34].

For these short distances of 1 mile and 1 km, men might profit from their higher skeletal muscle mass to swim faster than women. In the 26.4-km ‘Marathon Swim’ in Lake Zurich, Switzerland, men (42.0 ± 3.2 kg) had 12.6 kg more skeletal muscle mass than women (29.4 ± 2.9 kg) [24]. Another potential explanation could be the different numbers of women and men. The number of men competing in these events was considerably higher compared to the number of women. The ratio was 2.67 in ‘Ice Mile’ (123 men and 46 women) and 2.12 in ‘1 km Ice event’ (53 men and 25 women). Therefore, it is equally possible that this large difference may explain some of the observed high sex differences. However, we compared the sex difference between ‘Ice Mile’ and ‘1 km Ice event’ for the three and ten fastest swimmers. If/when this sport becomes more popular and more elite athletes start competing (especially women), the sex gap is likely to reduce. This is most likely the best explanation for sex differences. Future studies might then compare all women to all men to find the true sex difference without selection to the fastest swimmers. Indeed, the sex difference decreased in ‘Ice Mile’ from 16.9 % to 8.5 % when the ten fastest and all swimmers were considered, and from 21.1 % to 13.8 % in ‘1 km Ice event’ in the ten fastest and all swimmers (Table 1).

### No correlation between water temperature and swimming speed

A second important finding was that no correlation existed between water temperatures and swimming speed. Most probably the small range between nearly 0 and +5 °C gave no room for changes in swimming speed. Tipton et al*.* [35] investigated ten volunteers during three self-paced breaststroke swims in a variable-speed swimming flume at +25 °C, +18 °C, and +10 °C for a maximum of 90 min. Apart from oxygen consumption, rectal temperature, also swimming speed and angle, and stroke rate and length were measured. Swimming efficiency and length of stroke decreased more and rate of stroke and swim angle increased more in water of 10 °C than in warmer water.

### Limitations and implications for future research

A limitation of this study is the short time frame of 5 years and the low number of female swimmers. We assume that the sex difference will be lower with a higher number of successful women. Future studies should measure body fat in both women and men and body core temperature during the swims.

## Conclusion

In ice swimming in water colder than +5 °C, men were faster than women in 1 mile (‘Ice Mile’) and 1 km (‘1 km Ice event’). Between 2011 and 2015, the sex difference remained unchanged in ‘Ice Mile’ at ~18 %. In ‘Ice Mile’, calendar year, number of attempts, water temperature and wind chill showed no relation with swimming speed for women and men. For ‘Ice Mile’ and ‘1 km Ice event’, swimming speed showed no correlation to water temperature for women and men.
